# Study on the mechanism of lactic acid bacteria and their fermentation broth in alleviating hyperuricemia based on metabolomics and gut microbiota

**DOI:** 10.3389/fnut.2024.1495346

**Published:** 2024-12-04

**Authors:** Lijuan Rao, Biao Dong, Yanru Chen, Jiajing Liao, Chen Wang, Guiming Fu, Yin Wan

**Affiliations:** ^1^State Key Laboratory of Food Science and Resources, Nanchang University, Nanchang, Jiangxi, China; ^2^International Institute of Food Innovation Co., Ltd., Nanchang University, Nanchang, Jiangxi, China

**Keywords:** hyperuricemia, *Lactobacillus reuteri*, *Lactobacillus brevis*, metabolomics, gut microbiota

## Abstract

**Introduction:**

Hyperuricemia (HUA) is a metabolic disease caused by purine metabolism disorders in the body. Lactic acid bacteria (LAB) and their fermentation broth have the potential to alleviate hyperuricemia, but the potential mechanism of action is still unclear.

**Methods:**

The LAB with high inhibitory activity against xanthine oxidase (XOD) were screened out. Then the fermentation broth, fermentation supernatant and fermentation bacteria after fermentation of these LAB were administered into HUA mice, respectively.

**Results:**

*Lactobacillus reuteri* NCUF203.1 and *Lactobacillus brevis* NCUF207.7, of which fermentation supernatant had high inhibitory activity against XOD, were screened out and administered into HUA mice. Among them, *L. reuteri* strain, *L. reuteri* fermentation broth, *L. brevis* fermentation broth and *L. brevis* fermentation supernatant could significantly reduce serum uric acid levels and inhibited the liver XOD activity in HUA mice. The GC–MS metabolomics analysis of colon contents showed that supplementation of these four substances could partially reverse the down-regulation of energy metabolism pathways such as ketone body metabolism, pyruvate metabolism and citric acid cycle in HUA mice. It could also regulate amino acid metabolism pathways such as alanine metabolism, arginine and proline metabolism, glycine and serine metabolism, and repair the disorders of amino acid metabolism caused by HUA. In addition, the intervention of *L. brevis* fermentation broth and *L. brevis* fermentation supernatant may also accelerate the catabolism of uric acid in the intestine by up-regulating the urea cycle pathway. Fecal 16S rRNA sequencing analysis showed that their intervention increased the diversity of gut microbiota in HUA mice and alleviated the gut microbiota dysregulation caused by HUA.

**Discussion:**

These results indicated that the LAB and their fermentation broth may play a role in alleviating HUA by regulating intestinal metabolism and gut microbiota.

## 1 Introduction

Increased uric acid (UA) production or (and) decreased uric acid excretion in the body are the two main factors inducing hyperuricemia, and the evaluation standard for hyperuricemia is a serum uric acid (SUA) value higher than 7 mg/dL in men and 6 mg/dL in women (1). Hyperuricemia is associated with many metabolic diseases. Several large clinical studies have confirmed that hyperuricemia is a significant independent risk factor for hypertension (2), ischemic heart disease (3), and heart failure (4), as well as a causative factor for kidney disease, metabolic syndrome, and type 2 diabetes, with a linear dose–response relationship (5, 6).

In most vertebrates, UA is metabolized into allantoin by uricase and excreted out of the body. However, due to the lack of uricase in humans and primates, UA is the end product of purine metabolism (7). At physiological concentrations, UA acts as an antioxidant (8), but when the concentration of serum uric acid in human exceeds its saturation point, uric acid crystallizes and deposits in surrounding joints and tissues, causing gout or gouty arthritis (9). Xanthine oxidase (XOD) is a key enzyme in the production of uric acid, which can catalyze the transformation of hypoxanthine to xanthine, and then catalyze xanthine to produce UA, while accompanying reactive oxygen species (ROS) (10). Reducing UA levels in the body by inhibiting the activity of XOD is currently the main treating method, commonly use drugs such as allopurinol, febuxostat, and topiroxostat. However, these drugs can cause serious side effects on the body, such as Johnson syndrome, nephrotoxicity, hepatotoxicity, and allergic reactions such as toxic epidermal necrosis caused by allopurinol (11, 12). Therefore, it is of great significance to find safer and more effective natural uric acid lowering substances for the treatment of hyperuricemia.

About 70% of UA in the human body is excreted through the kidneys, while 30% is excreted through the intestines (13). The gut microbiota plays an undeniable role in the metabolism of UA in the intestines, which could involved in the metabolism of purines and uric acid and degrade UA into Short-chain fatty acid (SCFAs) such as acetic acid and butyric acid (14, 15). The gut microbiota of patients with hyperuricemia and gout was different from that of normal people. Liu et al. (15) have found that there is a widely distributed bacterial gene cluster in the gut microbiota that encodes a pathway for uric acid degradation, capable of metabolizing uric acid to xanthine or SCFAs. When gut microbiota is disrupted, intestinal metabolites such as SCFAs, amino acids, and trimethylamine produced by microorganisms tend to change. Studies have found a correlation between the fecal microbiome and metabolome of gout patients, which were changed at the same time. Changes in metabolites such as acetate, succinate, glucose, phenylalanine, glycine, aspartate are closely related to uric acid excretion and purine metabolism (16).

Probiotics are defined as “live microorganisms that, when administered in adequate amounts, confer a health benefit on the host” (17) and usually used as an additive in functional foods to improve gut microbiota and promote human health. Lactic acid bacteria (LAB), as a kind of probiotic, can improve the host’s physiological functions, strengthen the intestinal barrier, and exert immune effects (18). It has been shown that LAB can degrade nucleosides and inosine and reduce the SUA level in hyperuricemic mice (19, 20). *Limosilactobacillus fermentum* JL-3 strain isolated from “Jiangshui” with high degradation ability for UA has the effect of regulating gut microbiota dysbiosis caused by hyperuricemia (21). Some studies have found that the fermentation broth of LAB has the ability to inhibit XOD activity and can effectively reduce the level of SUA in hyperuricemic rats (22, 23). At present, most studies focus on the potential function of LAB strains to reduce UA and its regulatory effect on gut microbiota, while there are few studies on the effect and mechanism of LAB fermentation broth to alleviate hyperuricemia. Both LAB and their fermentation broth have the potential to improve hyperuricemia, but their mechanism of reducing UA remains to be further explored.

In this study, strains with high inhibitory activity against XOD were screened out from 16 strains of LAB. Then these strains and their fermentation broth were orally administered to hyperuricemic mice induced by potassium oxazinate and hypoxanthine to evaluate their uric acid lowering activity. Based on the analysis of metabolomics and gut microbiota, their potential mechanisms of uric acid lowering was investigated, which can provide a theoretical basis for the application of LAB and their fermentation broth in relieving HUA.

## 2 Materials and methods

### 2.1 Materials

Pyridine and bis(trimethylsilyl)trifluoroacetamide (BSTFA) were purchased from Xilong Science Co., Ltd., hypoxanthine and allopurinol were purchased from Shanghai Aladdin Biochemical Technology Co., Ltd., potassium oxyzincate, methoxyamine hydrochloride were purchased from Sigma Company, United States. Assay kits for the determination of uric acid (UA), creatinine (Cr), blood urea nitrogen (BUN) and XOD activity were obtained from Nanjing Jiancheng Biotechnology Institute (Nanjing, Jiangsu, China).

Sixteen LAB strains used in this study were preserved by our laboratory, which were selected from different samples (Table 1).

**Table 1:** Lactic acid bacteria strains used in this study.

Species	Number	Origin
*Lactobacillus plantarum*	NCUF201.4	Myrtle wine
*Lactobacillus acidophilus*	NCUF202.2	Myrtle enzyme
*Lactobacillus reuteri*	NCUF203.1	Milk curd
*Lactobacillus reuteri*	NCUF203.2	Milk curd
*Lactobacillus casei*	NCUF206.2	Sugar-lowering tea
*Lactobacillus brevis*	NCUF207.4	Milk curd
*Lactobacillus brevis*	NCUF207.7	Milk curd
*Lactobacillus harberi*	NCUF208.3	Cheese jerky
*Lactobacillus harberi*	NCUF208.4	Cheese jerky
*Lactobacillus harberi*	NCUF208.6	Cheese jerky
*Pediococcus pentosaceus*	NCUF209.1	Milk curd
*Pediococcus pentosaceus*	NCUF209.2	Milk curd
*Lactobacillus helveticus*	NCUF210.1	Milk curd
*Lactobacillus fermentum*	NCUF211.1	Cheese jerky
*Enterococcus faecalis*	NCUF212.1	Intestinal Digestive Promoters
*Pediococcus acidilactici*	NCUF213.1	Cheese jerky

### 2.2 The inhibitory activity of lactic acid bacteria fermentation supernatant on XOD

The 16 activated strains of LAB were cultured in liquid de Man Rogosa-Sharpe (MRS) medium at 37°C for 24 h, respectively. When the bacterial density reached 2 × 10^9^ CFU/mL, the culture solution was centrifuged at 8,000 rpm and 4°C for 10 min to remove bacterial cells and obtain the fermentation supernatant.

In a 1 mL sodium pyrophosphate buffer solution (0.2 mol/L, pH 8.0) system, 20 μL lactic acid bacteria fermentation supernatant and 200 μL XOD (0.1 u/mL) were firstly added to 480 μL of sodium pyrophosphate buffer, 37°C water bath for 15 min. The reaction was initiated by adding 200 μL xanthine solution, and lasted for 3 min. The absorbance values of uric acid for 0 and 3 min were measured by spectrophotometer at 290 nm, respectively. The calculation formula was as follows (22):


$$XOI\;\left(\%\right)=\left(1-\frac{{OD}_{\text{after}}-{OD}_{\text{before}}}{{\text{BlankOD}}_{\text{after}}-{\text{BlankOD}}_{\text{before}}}\right)\times 100\%\text{.}$$

XOI is the inhibition rate of XOD, OD_after_ is the absorbance value of the experimental group at 3 min of reaction, OD_before_ is the absorbance value of the experimental group at 0 min, BlankOD_after_ is the absorbance value of the blank group at 3 min, and BlankOD_before_ is the absorbance value of the blank group at 0 min.

### 2.3 Animal experiments

#### 2.3.1 Preparation of lactic acid bacteria and their fermentation broth

The selected LAB strains were cultured in MRS broth at 37°C for 24 h to obtain the fermentation broth with a cell density of 2 × 10^9^ CFU/mL. Then, the fermentation broth was centrifuged at 8,000 rpm for 10 min, and the supernatant was passed through a 0.22 μm filter to obtain the sterile fermentation supernatant (23). The bacterial precipitate was washed with normal saline and resuspended in normal saline to obtain the bacterial suspension of 2 × 10^9^ CFU/mL. Freshly prepared fermentation broth samples, fermentation supernatant samples and bacterial suspension samples were orally administered to mice as soon as possible.

#### 2.3.2 Animal experiment grouping and administration

The SPF-grade male Kunming mice (20 ± 2 g) were purchased from Hunan Slaughter Jingda Laboratory Animal Co., Ltd. (Certificate No. SCXK (Xiang) 2016–0002). All mice were fed with standard chow and water, housed at 20 ± 2°C in 12-h light/dark cycles and the relative humidity of the animal room was 40–60%.

**Figure 1:**
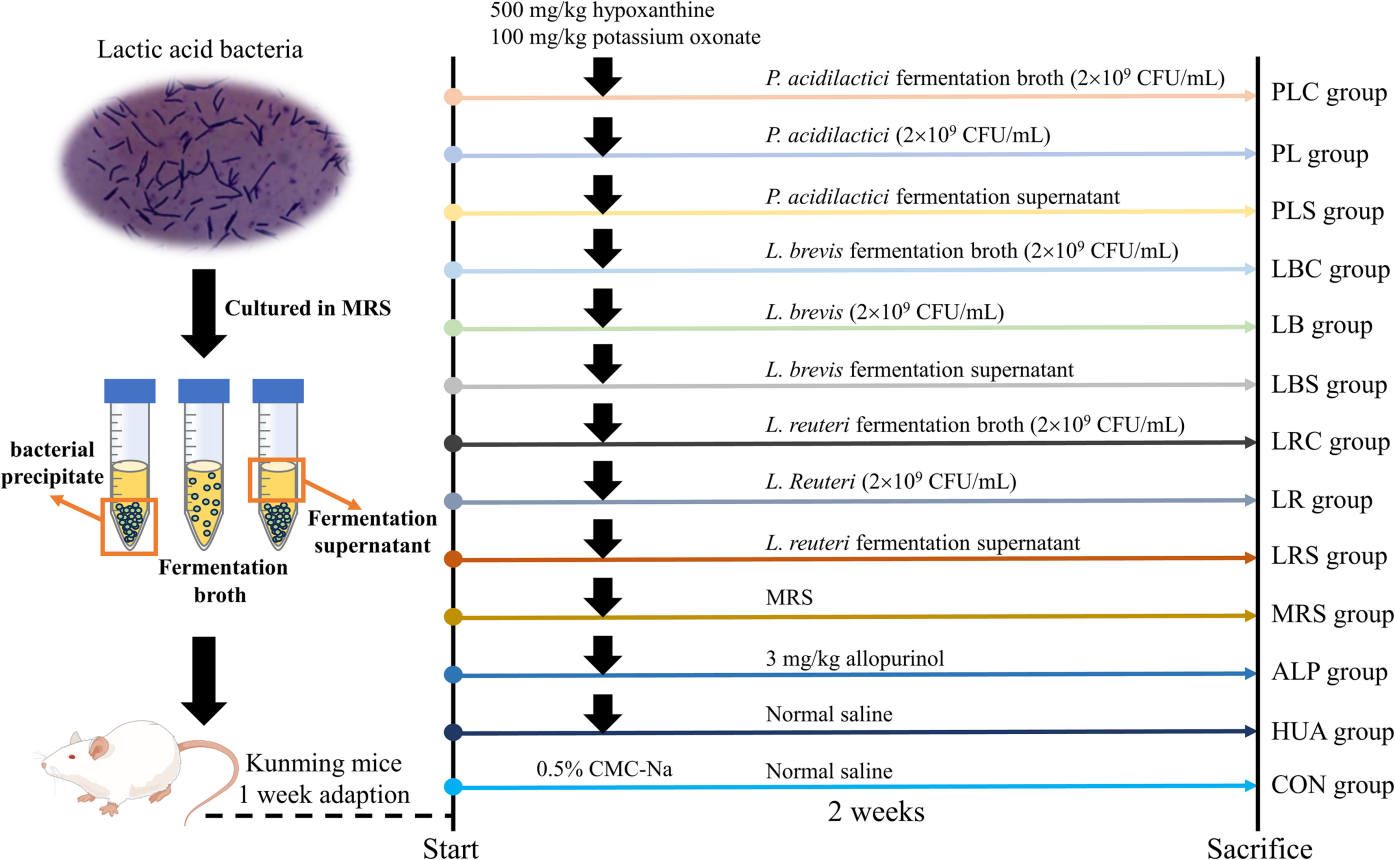
Experimental chart of lactic acid bacteria and their fermentation broth in treatment of HUA mice.

After 1 week of domestication, the mice were randomly divided into 13 groups (*n* = 8): control group (CON group), hyperuricemia model group (HUA group), allopurinol group (ALP group), *L. reuteri* group (LR group), *L. brevis* group (LB group), *P. acidilactici* group (PL group), MRS medium group (MRS group), *L. reuteri* fermentation supernatant group (LRS group), *L. brevis* fermentation supernatant (LBS group), *P. acidilactici* fermentation supernatant group (PLS group), *L. reuteri* fermentation broth group (LRC group), *L. brevis* fermentation broth group (LBC group), *P. acidilactici* fermentation broth group (PLC group). The establishment of the HUA model was shown in Figure 1. The CON group was gavaged with 0.5% sodium carboxymethyl cellulose (CMC-Na) solution, and other groups were gavaged with hypoxanthine (500 mg/kg) and potassium oxonate (100 mg/kg) dissolved in 0.5% CMC-Na solution to construct a model of hyperuricemia. After 1 h of modeling, the ALP group was administered 5 mg/kg allopurinol, and the other experimental groups were gavaged with 0.1 mL/10 g body weight of sample solution.

Each mouse was weighed and observed every day. On the 15th day, the mice were fasted for 12 h and anesthetized with pentobarbital sodium. Blood, liver, colon and cecum of mice were collected and stored at −80°C for further biochemical analysis.

### 2.4 Determination of serum and liver biochemical indicators

The collected blood samples were centrifuged at 3,000 r/min for 10 min after 2 h and the obtained supernatant was serum. The levels of UA, creatinine (Cr), and blood urea nitrogen (BUN) in serum and XOD activity in liver were detected by commercially available kits.

### 2.5 The gas chromatography–mass spectrometry (GC–MS) analysis of colon metabolites

The GC–MS conditions were referred to Gao et al. (24) and made slight modifications as follows: 100 mg of mice colon contents were weighted and extracted with 1 mL methanol. The mixture was vortexed and sonicated for 10 min, then held on ice for 30 min. Six hundred microliter of the supernatant was dried with a nitrogen blower, then added 60 μL 20 mg/mL pyridine solution of methoxylamine hydrochloride to the dried sample, and finally added 60 μL BSTFA for reaction at 70°C for 1 h. All samples were combined as quality control (QC) samples, and the samples were subjected to blind and randomized treatment for machine testing. Deionized water was used as the control sample.

The GC–MS conditions were as follows: HP-5MS capillary column (Agilent J&W Scientific, Folsom, California, United States), 20:1 split ratio, sample volume 1 μL, 1.0 mL/min constant flow rate helium as carrier gas. The temperatures of injector, transfer line, and electron impact (EI) ion source were set to 280, 250, and 150°C, respectively. The initial oven temperature was held at 70°C for 2 min, ramped to 280°C at a rate of 10°C/min for 5 min. The electron energy was 70 ev, and mass data was collected in a full scan mode (m/z 50–800).

The XCMS software package of R software was used for peak selection and retention time correction of the data, and then the preprocessed data was normalized in R. The QC samples were used to calculate the relative standard deviation (RSD) value, selecting RSD < 30% of the characteristic peaks were used for subsequent multivariate analysis. The fragmentation pattern of mass spectrometry of the detected metabolite were compared with the NIST database to determine the material structure of the small molecule. The enrichment analysis of metabolic pathways was performed using MetaboAnalyst 4.0.

### 2.6 16S rRNA gene sequencing

After extracting the total DNA of the fecal sample, PCR amplification was performed and the product was purified. IlluminaHiSeq2500 was used for sequencing to obtain Raw Reads. Using Trimmomatic v0.33 software to filter the sequenced Raw Reads, and then used cutadapt 1.9.1 software to identify and remove primer sequences to obtain high-quality Reads without primer sequences, barcodes, and interfaces. Splicing high-quality Reads and removing chimeras.

### 2.7 Statistical analysis

Data were expressed as the mean ± standard deviation. Statistical analysis was performed using R 3.6.3 and the IBM SPSS Statistics 21. The statistical difference between the control and experimental groups was analyzed using one-way analysis of variance (ANOVA). *p*-value<0.05 was considered statistically significant.

## 3 Results

### 3.1 Effects of lactic acid bacteria fermentation supernatant on XOD activity

**Figure 2:**
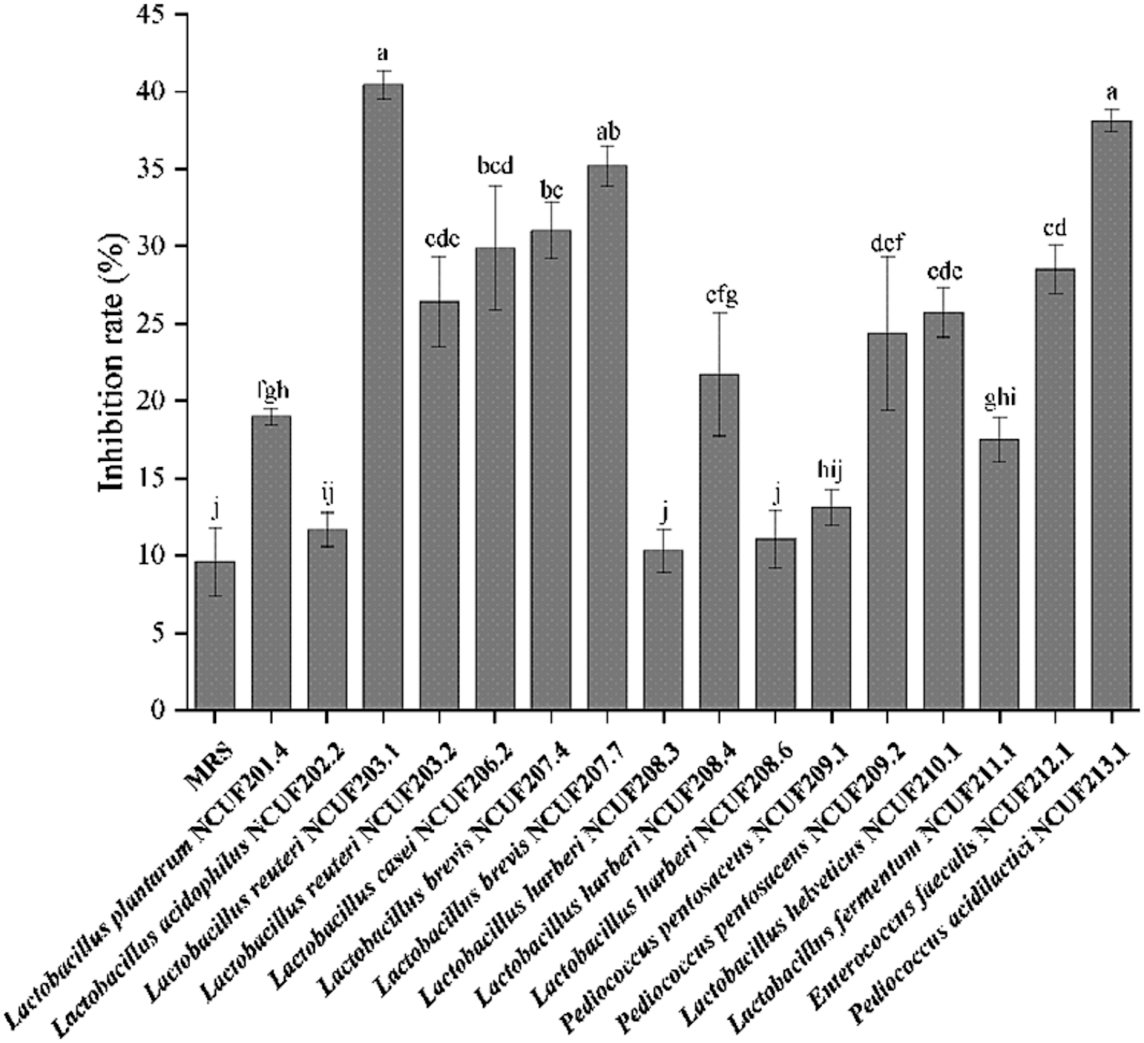
Inhibition rate of xanthine oxidase (XOD) by fermentation supernatant of lactic acid bacteria. Different letters indicate significant differences between groups (*p* < 0.05).

The XOD inhibition experiment results *in vitro* showed that the fermentation supernatant of various lactic acid bacteria had an inhibitory effect on XOD (Figure 2). Compared with MRS, the fermented supernatant of LAB showed better XOD inhibition rate. Among them, the fermentation supernatant of *L. reuteri* NCUF203.1 had the best inhibitory effect on XOD, followed by the fermentation broth of *P. acidilactici* NCUF213.1 and *L. brevis* NCUF207.7, with inhibitory rates of 40.46, 38.15, and 35.24%, respectively, both significantly higher than MRS (*p* < 0.05) (Figure 2).

### 3.2 Effects of lactic acid bacteria and their fermentation broth on the body weight, serum uric acid levels, serum creatinine levels, serum urea nitrogen levels and hepatic XOD activity of HUA mice

**Figure 3:**
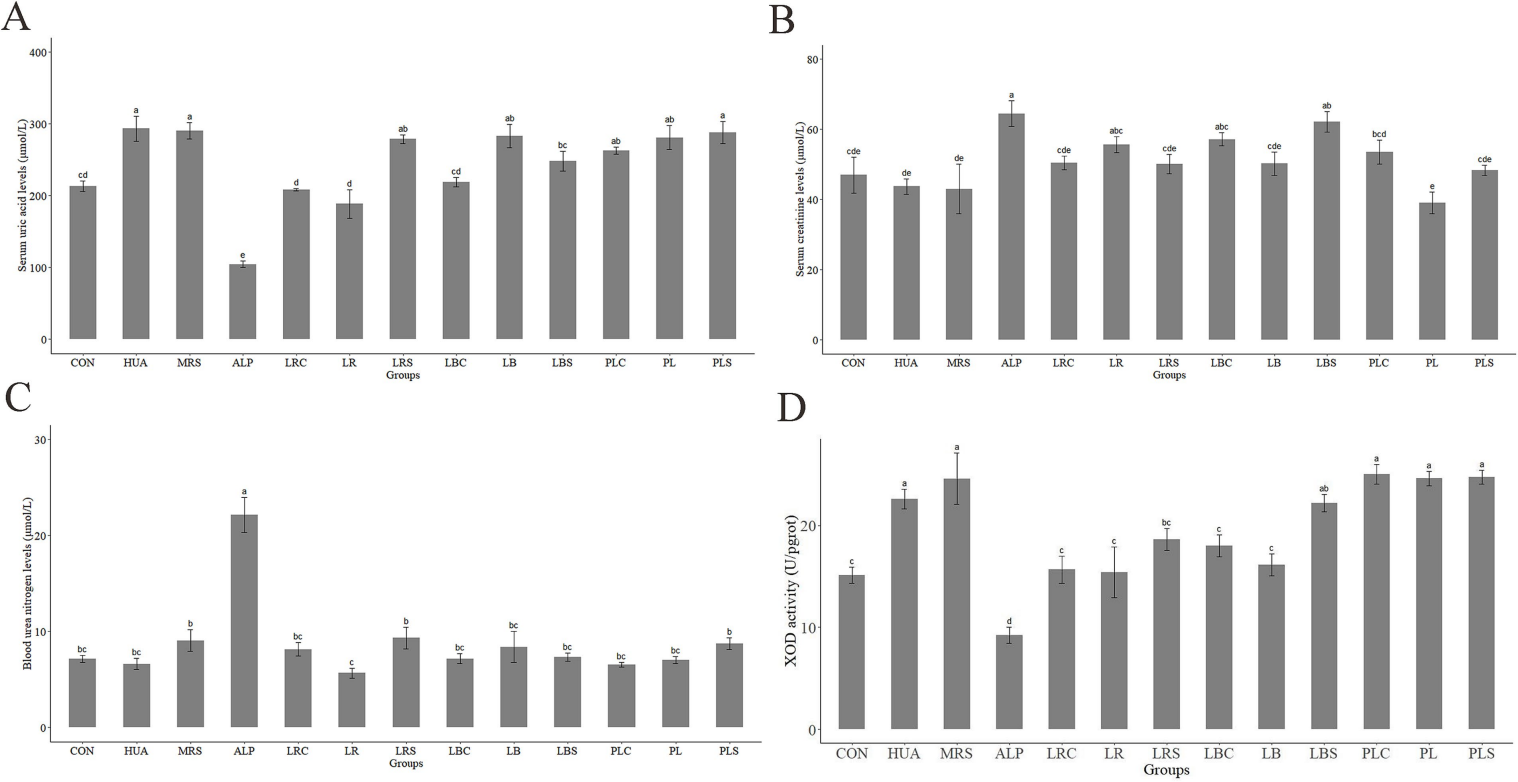
Changes of serum uric acid (A), serum creatinine (B), serum urea nitrogen (C) and hepatic xanthine oxidase activity (D) of mice. Different letters indicate significant differences between groups (*p* < 0.05).

It can be observed that during the two-week animal experiment, except for the ALP group, the weight of other mice generally showed an upward trend over time (Supplementary Table S1). The weight growth rate of mice in the HUA group was not significantly different from that of the CON group. However, the weight gain in ALP group showed a negative growth rate, which was significantly lower than that in the HUA groups (*p* < 0.05). Compared with the HUA group, the weight growth rate of mice in each intervention group of LAB decreased. As shown in Figure 3A, the serum uric acid level of mice in the HUA group was remarkably higher than that of the CON group by 37.67% (*p* < 0.01). Compared with the HUA group, intragastric administration of MRS had no significant effect on SUA in mice. The SUA levels of mice decreased in the LRS, LB, PL, PLC, and PLS groups compared to the HUA group, but there was no significant difference. However, it was significantly reduced in the LRC, LR, LBC, and LBS groups (*p* < 0.05).

The serum creatinine and urea nitrogen levels in the ALP group were the highest among the groups, at 64.53 and 22.17 μmol/L, respectively, which were significantly increased compared to the CON group (*p* < 0.05, Figures 3B,C). There was no significant difference in serum creatinine and urea nitrogen levels between the CON group and each other groups. As shown in Figure 3D, it was found that the XOD activity in the HUA group was significantly increased to 22.57 U/pglot compared with that of the CON group (*p* < 0.05). The XOD activity was no significant difference between the MRS, PLC, PL, and PLS groups compared with the HUA group, but it was significantly reduced in the LR, LRC, LRS, LBC, and LB groups (*p* < 0.05).

### 3.3 Effects of lactic acid bacteria and their fermentation broth on intestinal metabolism in HUA mice

**Figure 4:**
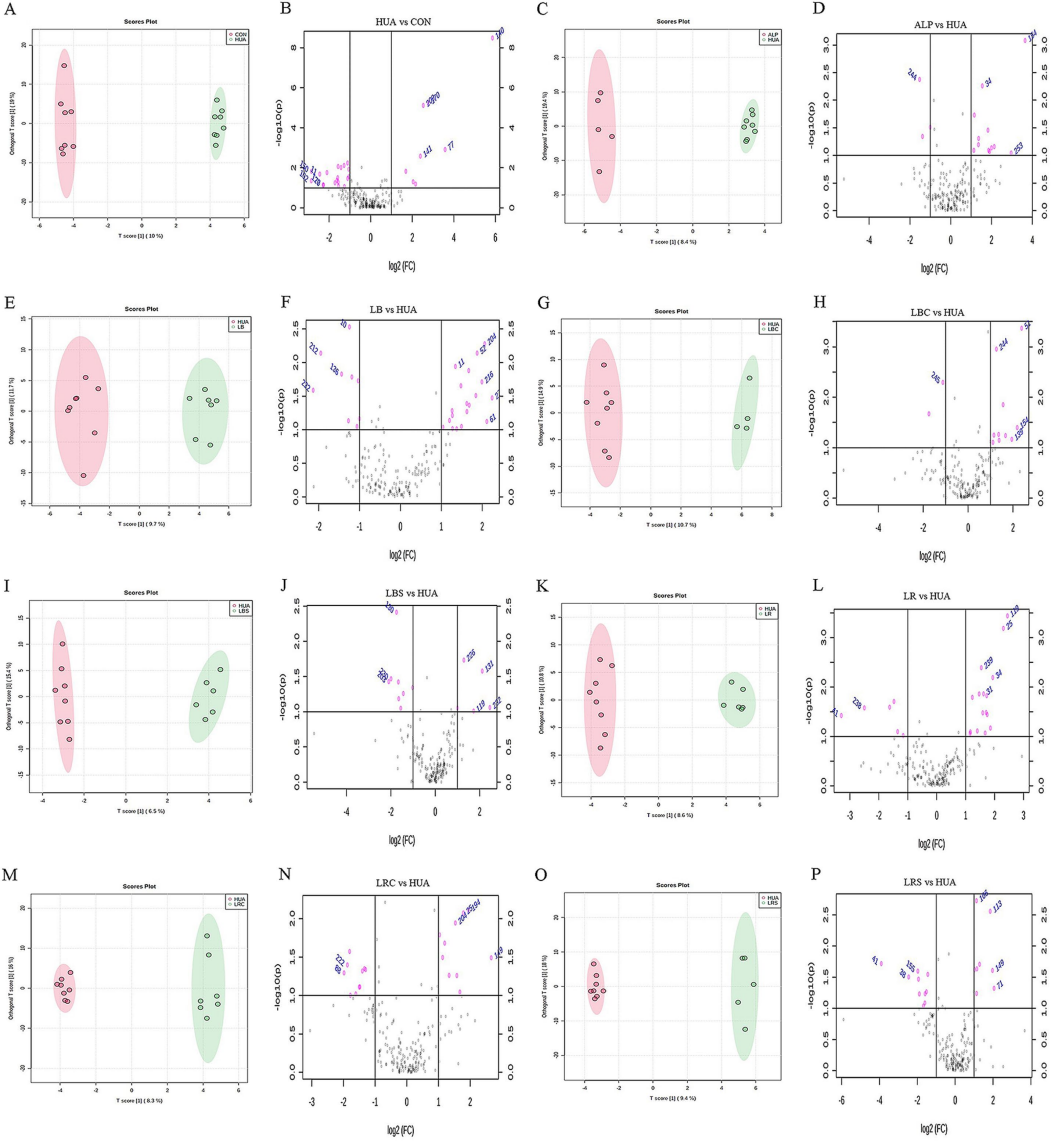
Obtaining differential metabolites based on multivariate data analysis (A,C,E,G,I,K,M,O were OPLS-DA score chart; B,D,F,H,J,L,N,P were volcanic maps).

The results of animal experiments in Figure 3 showed that the supplementation of *L. reuteri*, *L. brevis* and their fermentation broth could reduce the level of SUA in hyperuricemic mice, while supplementation of *P. acidilactici* and its fermentation broth had no significant effect on it. Therefore, the metabolomics analysis on the colon contents of mice in each intervention groups of *L. reuteri* and *L. brevis* were conducted to further explore their mechanism of lowering serum uric acid levels. The RSD value of the computed quality control sample (QC) was used to evaluate the data quality of GC–MS. As shown in Supplementary Figure S1, the proportion of characteristic peaks with RSD value less than 30% was greater than 75%, indicating that the sequencing data was generally good, and characteristic peaks with RSD < 30% were selected for subsequent analysis. Orthogonal partial least squares discriminant analysis (OPLS-DA) was used to compare and analyze the multivariate data between each experimental group and HUA group. Figures 4A,C,E,G,I,K,M,O showed that there were obvious separation between each two groups, and there were significant differences in metabolites between HUA group and other groups. In order to further analyze the metabolites in the eight groups, the fold change and *p*-value of metabolites between the groups were calculated and plotted as volcanoes, as shown in Figures 4B,D,F,H,J,L,N,P. In the volcano map, the left side of the *X*-axis was down-regulated metabolites, and the right side was up-regulated metabolites. The gray part represented the metabolites with no difference in comparison between the two groups.

The up-regulation and down-regulation of differential metabolites between the two groups were shown in Supplementary Table S2. After 14 days of HUA modeling, the content of intestinal metabolites in hyperuricemic mice such as uracil, dihydrouracil, purine, xanthine, and inosine were up-regulated, while metabolites such as succinic acid, l-phenylalanine, stearic acid, l-arginine, uridine, l-glycine were down-regulated. Compared with the HUA group, the up-regulated metabolites in the LR group included succinic acid, l-phenylalanine, stearic acid, l-threonine, l-alanine, sarcosine and phenylpyruvic acid, but metabolites such as uridine, l-glycine, and l-valine were down-regulated. l-glycine, *β*-sitosterol, phenylpyruvate, and l-serine were up-regulated in the LRC group, while metabolites such as creatinine, dihydrouracil, d-malic acid, and 2-hydroxytyrosol were down-regulated. In the LRS group, metabolites such as stearic acid, l-glycine, l-methionine, succinic acid, l-glutamic acid, and l-lysine were up-regulated, while metabolites such as l-leucine, creatinine, purine, l-valine, sarcosine, l-serine, and trehalose were down-regulated. After treating hyperuricemic mice with *L. brevis*, the content of urea, l-tyrosine, stigmasterol and other metabolites increased in the LB group, while succinic acid, l-leucine, inosine, 3-methyladenine, *β*-sitosterol and other metabolites decreased. Metabolites such as urea, l-glycine, l-tyrosine, *β*-sitosterol and l-threonine increased, while the content of d-mannose decreased in the LBC group. In the LBS group, the content of metabolites such as urea, stearic acid, dihydrouracil and l-cysteine increased and the metabolites of uridine, xanthine, l-alanine, d-malic acid, l-proline decreased.

**Figure 5:**
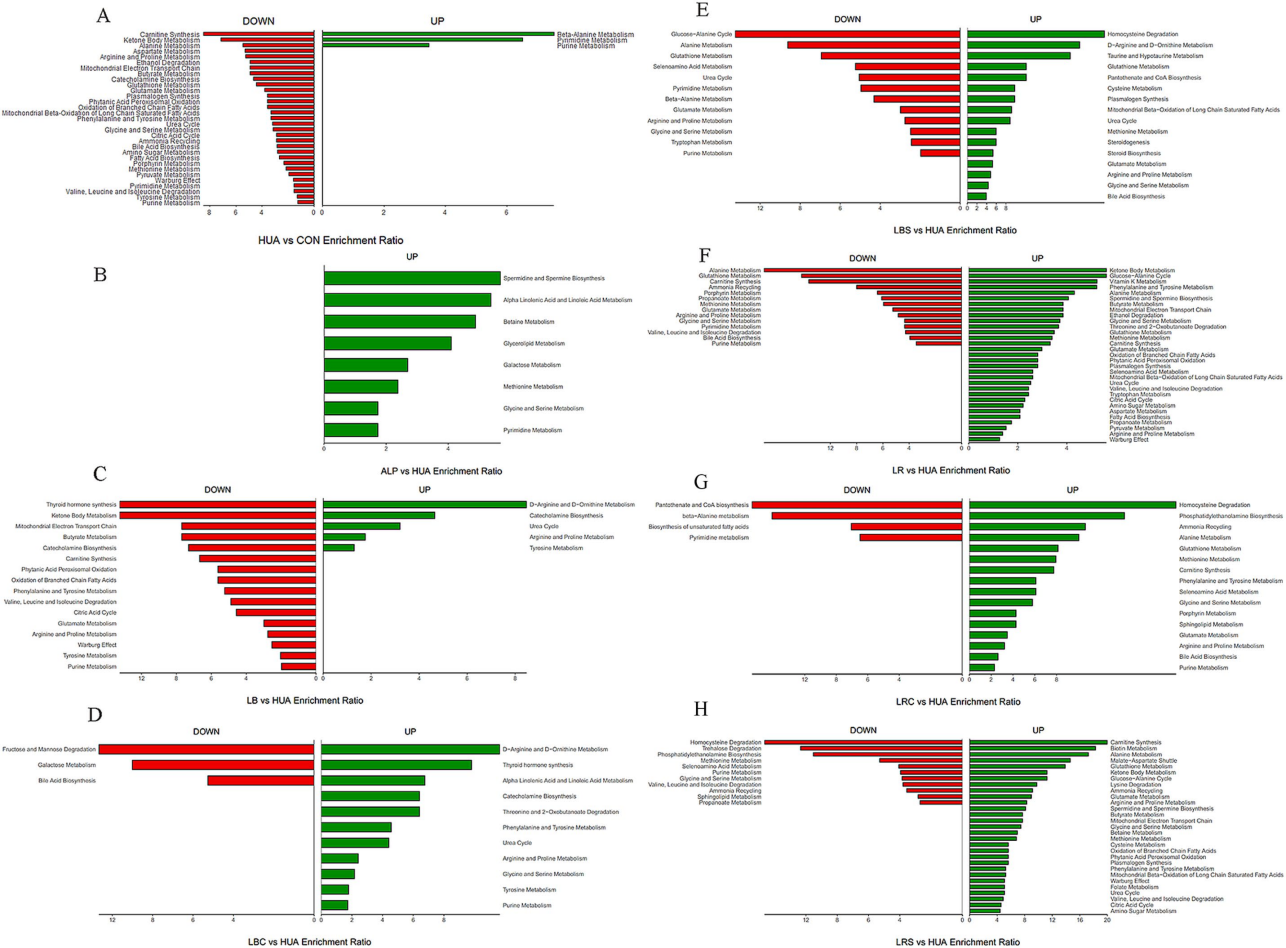
Column diagram of metabolite enrichment analysis. Panel (A) was the metabolic pathways changed in HUA group compared with CON group; Panels (B–H) were the metabolic pathways changed in ALP group, LB group, LBC group, LBS group, LR group, LRC group and LRS group compared with HUA group, respectively.

In order to further elucidate the potential mechanism of the regulation of differential metabolites mentioned above, KEGG database was used to enrich and analyze the important metabolic pathways in different metabolites to analyze the mechanism of the effects of *L. reuteri*, *L. brevis* and their fermentation broth on the metabolism of hyperuricemia mice. As shown in Figure 5, it was observed that the beta-alanine metabolism, pyrimidine metabolism and purine metabolism pathways were up-regulated in the HUA group, metabolic pathways such as carnitine synthesis, alanine metabolism, aspartate metabolism, arginine and proline metabolism, phenylalanine and tyrosine metabolism, glycine and serine metabolism, butyrate metabolism, glutathione metabolism and urea cycle were down-regulated, and energy metabolism pathways such as ketone body metabolism, citric acid cycle, pyruvate metabolism were also down-regulated compared with those of in the CON group. The intervention of *L. reuteri* and its fermentation broth upregulated metabolism pathways such as alanine metabolism, phenylalanine and tyrosine metabolism, glycine and serine metabolism, and carnitine synthesis compared with the HUA group. At the same time, supplementing *L. reuteri* could partially up-regulated energy metabolism pathways such as ketone body metabolism and citric acid cycle. The purine metabolism pathway was down-regulated in both the LR and LRS groups. The intervention of *L. brevis* and its fermentation broth upregulated the metabolism pathways such as d-arginine and d-ornithine metabolism, arginine and proline metabolism, and urea cycle, while purine metabolic pathway was down-regulated in both LB and LBS groups.

### 3.4 Effects of lactic acid bacteria and their fermentation broth on the composition of gut microbiota in HUA mice

**Figure 6:**
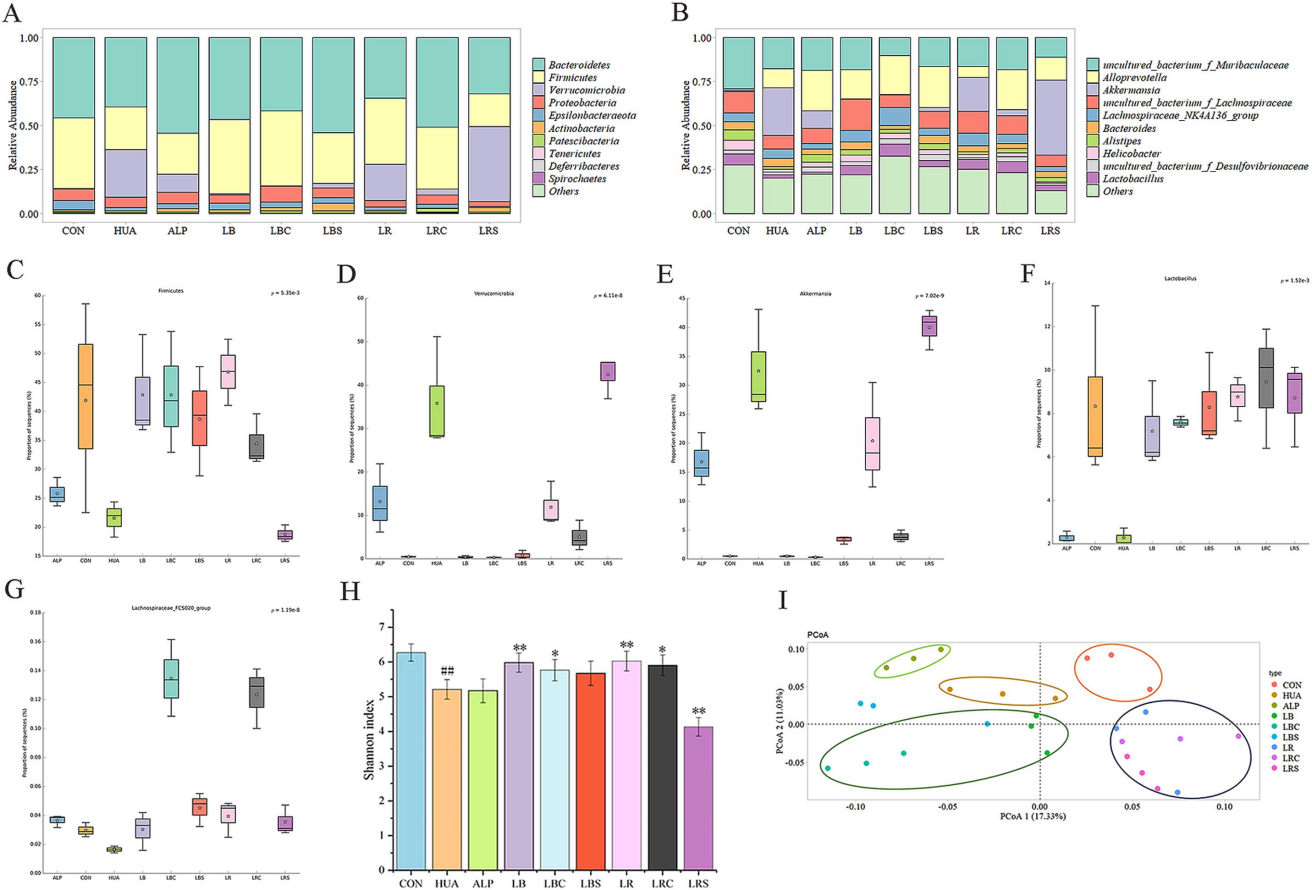
Effects of lactic acid bacteria and their fermentation broth on the gut microbiota of hyperuricemic mice. (A) Species annotation by phylum level. (B) Species annotation by genus level. (C) The relative abundance of *Firmicutes*. (D) The relative abundance of *Verrucomicrobia*. (E) The relative abundance of *Akkermansia*. (F) The relative abundance of *Lactobacillus*. (G) The relative abundance of *Lachnospiraceae_FCS020_group*. (H) *α* diversity indicated by Shannon index. (I) *β* diversity based on PCoA analysis. (#*p* < 0.05, ##*p* < 0.01, compare with CON group, *p < 0.05, **p < 0.01, compare with HUA group).

The association between the intake of *L. reuteri*, *L. brevis* and their fermentation broth and the changes of gut microbiota was evaluated at the phylum and genus levels. At the phylum level, *Bacteroidetes*, *Firmicutes*, *Verrucomicrobia*, *Proteobacteria*, *Epsilonbacteraeota*, and *Actinobacteria* constituted the main microbiota, among which *Bacteroidetes* and *Firmicutes* were dominant (Figure 6A). The STAMP software was used to analyze the differential flora between groups. Through the comparisons of multiple groups, there were significant differences in *Firmicutes* (*p* = 5.35e-3) and *Verrucomicrobia* (*p* = 6.11e-8) at the phylum level. At the genus level, *Akkermansia* (*p* = 7.02e-9), *Lactobacillus* (*p* = 1.52e-3) and *Lachnospiraceae_FCS020_group* (*p* = 1.19e-8) had a significant difference. It was found that compared with the CON group, the relative abundance of *Firmicutes* in the HUA group was significantly reduced, and the relative abundance of *Verrucomicrobia* was markedly increased. However, the intervention groups of *L. reuteri* (LR, LRC) and *L. brevis* (LB, LBC, and LBS) showed a significant increase of *Firmicutes* and a significant decrease of *Verrucomicrobia* compared with the HUA group (Figures 6C,D).

At the genus level, the main dominant genera of gut microbiota in mice were uncultured_bacterium_f_Muribaculaceae, Alloprevotella, Akkermansia, uncultured_bacterium_f_Lachnospiraceae, Lachnospiraceae_NK4A136_group, Bacteroides, Alistipes, Helicobacter, uncultured_bacterium_f_Desulfovibrionaceae and Lactobacillus (Figure 6B). Except for the LRS group, other lactic acid bacteria intervention groups were able to reverse the increase in the relative abundance of Akkermansia and the decrease in the relative abundance of Lactobacillus and Lachnospiraceae_FCS020_group in HUA mice (Figures 6E–G).

The Shannon index was used to evaluate the gut microbiota alpha diversity (Figure 6H). It was shown that the gut microbiota diversity of HUA group was significantly reduced compared with that of the CON group, and the treatment of allopurinol also reduced the gut microbiota diversity. Except for the LRS group, Shannon index of LB (*P* < 0.01), LBC (*P* < 0.05), LR (*P* < 0.01), LRC (*P* < 0.05) groups was significantly higher than that of HUA group. The PCoA (Principal component analysis) of Weighted Unifrac distance reflected beta diversity, indicating there were differences in gut microbiota composition between groups (Figure 6I).

**Figure 7:**
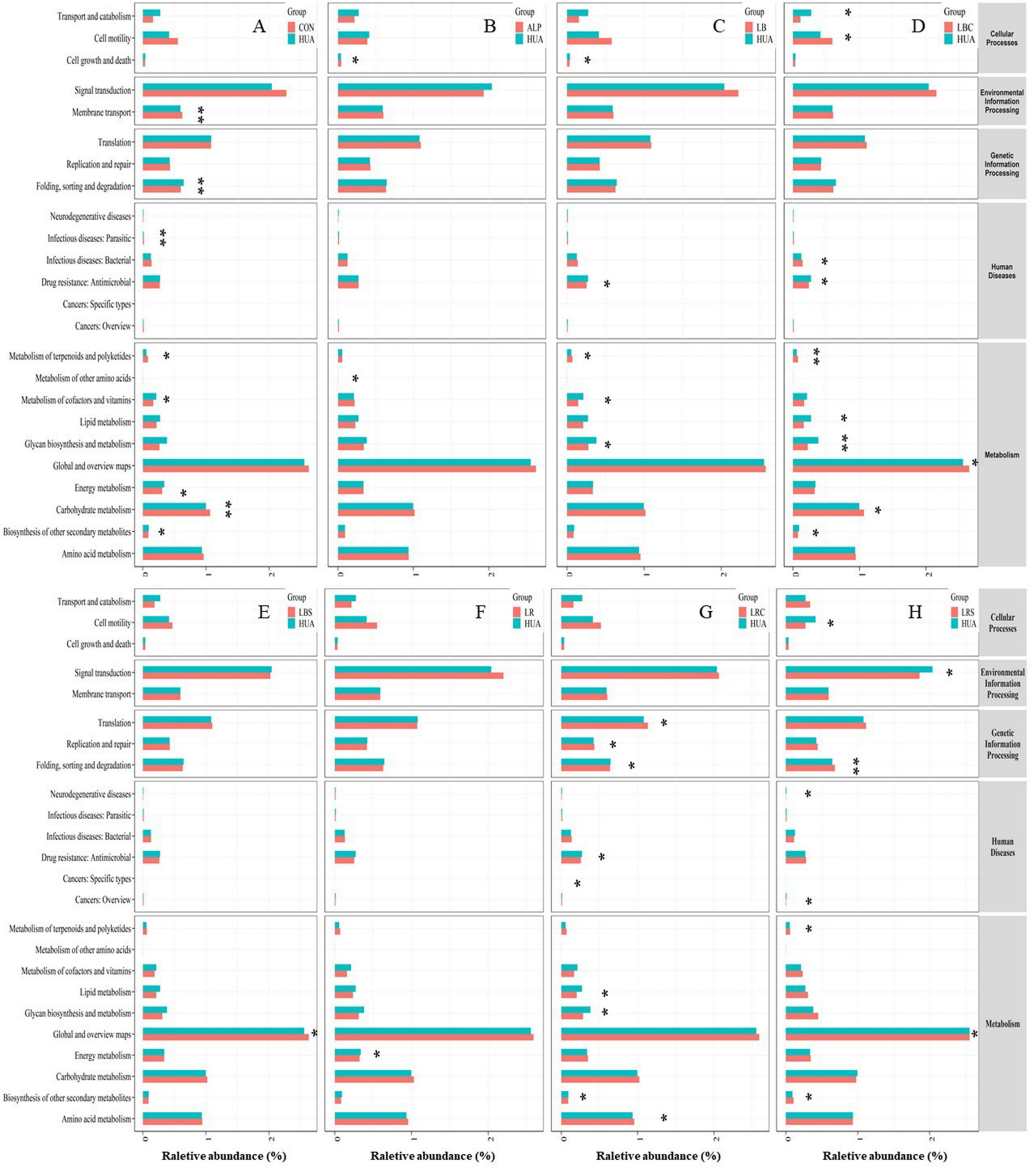
Difference analysis of KEGG metabolic pathway in gut microbiota of mice. Panel (A) was the metabolic pathways changed in HUA group compared with CON group; Panels (B–H) were the metabolic pathways changed in ALP group, LB group, LBC group, LBS group, LR group, LRC group and LRS group compared with HUA group, respectively (*p < 0.05, **p < 0.01).

The potential metabolic function of the gut microbiota will be caused by changes in microbiota structure. PICRUSt2 software was used to predict the gene function, and the gut microbiota function was analyzed based on the KEGG database (Figure 7). The results showed that the carbohydrate metabolism and metabolism of terpenoids and polyketides the HUA group was down-regulated, and the energy metabolism, cofactor and vitamin metabolism and biosynthesis of other secondary metabolites were up-regulated compared with the CON group. After the intervention of *L. reuteri*, the energy metabolism pathway of HUA mice was significantly down-regulated (*p* < 0.05), while the lipid metabolism, sugar biosynthesis and metabolic pathways were significantly down-regulated and the amino acid metabolism pathway was significantly up-regulated after oral administration of *L. reuteri* fermentation broth (*p* < 0.05). Supplementation of *L. brevis* fermentation broth in HUA mice significantly reversed the down-regulation of metabolism of terpenoids and polyketones induced by HUA (*p* < 0.01), while lipid metabolism and glucose biosynthesis and metabolic pathways were significantly down-regulated (*p* < 0.05). Compared with the HUA group, only the global and overview maps metabolic pathway were significantly down-regulated in LBS group (*p* < 0.05).

## 4 Discussion

When the purine metabolism in the body is disordered or excessive intake of purine substances, the body will produce a large amount of uric acid and distribute it in the blood, which leads to hyperuricemia and brings harm to the body. XOD is the key enzyme that catalyzes the production of uric acid from hypoxanthine and xanthine (25). It is mainly distributed in the liver and intestines and highly active XOD can easily cause the body to produce a large amount of uric acid (26). By inhibiting XOD activity, the production of uric acid in the human body can be reduced to prevent and treat hyperuricemia. Among the 16 strains of LAB, the fermentation supernatant of *L. reuteri* and *L. brevis* had a good effect on inhibiting XOD activity. Chen also found that the microbial fermentation supernatant could inhibit XOD activity (22), and there may be small molecules in the fermentation supernatant that inhibit XOD activity.

In this study, hypoxanthine combined with potassium oxyzincate were used to establish hyperuricemia model. The serum uric acid in the HUA group reached 293.43 μmol/L after 2 weeks, which proved the effectiveness of this method. Supplementing *L. reuteri* and its fermentation broth could significantly reduce the serum uric acid level of the HUA mice, but the effect of *L. reuteri* fermentation supernatant was not obvious, which indicated that *L. reuteri* bacteria may play a major role in lowering uric acid rather than the metabolites produced by it. Kuo et al. (27) also found that the level of SUA in HUA rats was significantly reduced by 60% after supplementing with *L. reuteri* TSR332, and no obvious side effects were observed. On the contrary, the intervention of *L. brevis* fermentation broth and *L. brevis* fermentation supernatant had better uric acid lowering effect than those of *L. brevis*, and could significantly reduce the serum uric acid level of HUA mice, indicating that the uric acid-lowering effect was mainly contributed by the metabolites produced by *L. brevis*, rather than the bacteria itself. The study have also found that *Lactobacillus* MFE-21 fermentation broth had a good uric acid-lowering effect on HUA mice (22). CR and BUN are important indicators to evaluate kidney damage (28). Our study showed that compared with the CON group, there was no significant increase in the levels of CR and BUN in other groups except for the ALP group, which indicated that 2 weeks of modeling did not cause significant damage to the kidneys of mice, and the intervention of the two lactic acid bacteria cells and their fermentation broth did not damage the kidneys of mice. Allopurinol, a drug clinically used to treat hyperuricemia, is prone to adverse side effects after long-term use. The levels of serum CR and BUN of the ALP group were significantly increased, causing serious damage of allopurinol to the kidney. It was the same as the results of Zhou et al. (29). The intervention of *L. reuteri*, *L. reuteri* fermentation broth and *L. brevis* fermentation broth could significantly reduce the XOD activity in the liver of HUA mice and reduce the production of serum uric acid, thereby alleviating the disease.

It was reported that the metabolism of hyperuricemia and gout patients was disordered compared with normal people, and the metabolites were changed (30). In order to further investigate the mechanism of reducing uric acid by lactic acid bacteria and their fermentation broth, we used non-targeted metabolomics was used to detect the differences of intestinal metabolites between groups. Results showed that the contents of metabolites such as purine, xanthine and inosine, the precursors to uric acid, were increased in the HUA group, and the purine metabolic pathway was up-regulated, thereby increasing the production of uric acid. At the same time, the metabolites such as succinic acid, l-phenylalanine, l-arginine, and l-glycine decreased, and amino acid metabolism and energy metabolism were disturbed, which was similar to the findings of Shen et al. (30).

The metabolic biomarkers in HUA rats are mainly involved in purine metabolism, arginine and proline metabolism, citric acid cycle, phenylalanine metabolism, tryptophan metabolism and glycerophospholipid metabolism (31). After intervention with *L. reuteri*, the levels of down-regulated metabolites such as succinic acid, l-phenylalanine, stearic acid and l-glycine were increased in HUA mice, and the energy metabolic pathways such as ketone body metabolism and citric acid cycle were up-regulated, which could alleviate the disturbance of energy metabolism caused by HUA. Succinic acid is involved in energy metabolism and can regulate the intestinal-immune tissue axis by activating intestinal mucosal cells, as well as regulating intestinal homeostasis (32). Tan et al. (33) found that phenylalanine is important in the interaction between Lesinurad and other URAT1 inhibitors, which may be part of the inhibitor binding site in the URAT1 transporter channel, and URAT1 is responsible for the reabsorption of uric acid. Supplementing with glycine can promote the excretion of uric acid in the body, and significantly increase the clearance rate of urate salts (34). The increase of these metabolites in the LR group could regulate intestinal homeostasis, reduce uric acid reabsorption and promote uric acid excretion. Supplementation of the *L. reuteri* fermentation broth could reduce the content of creatinine and repair kidney damage caused by hyperuricemia. Both interventions upregulated alanine metabolism, phenylalanine and tyrosine metabolism, glycine and serine metabolism pathways. Among them, the alanine metabolism pathway was related to urate metabolism (35), and glycine and serine metabolism can affect the antioxidant capacity of cells and regulate the synthesis of glutathione (36), indicating that oral administration of *L. reuteri* and its fermentation broth could regulate amino acid metabolism disorders caused by HUA, regulate urate metabolism, and enhance the body’s antioxidant capacity.

The intervention of the *L. brevis* fermentation broth and the fermentation supernatant of *L. brevis* both increased the content of urea, the end product of purine metabolism, and upregulated the urea cycle pathway, indicating that the metabolism of purine was accelerated, thereby accelerating the catabolism of uric acid in the intestine. At the same time, the two interventions reversed the down-regulation of amino acid metabolism pathways such as arginine and proline metabolism, glycine and serine metabolism caused by HUA. In addition, supplementing *L. brevis* fermentation supernatant could also partially reverse the changes of metabolites such as l-glycine and stearic acid caused by hyperuricemia, decrease the content of metabolites such as xanthine, and downregulate the purine metabolism pathway, which can further reduce the production of uric acid.

The change of gut microbiota is closely related to hyperuricemia. More and more evidences show that gut microbiota was not only related to the pathogenesis of disease, but also to the effectiveness of disease treatment (37). In our study, the gut microbiota of hyperuricemia mice was significantly changed. Administration of *L. reuteri*, *L. reuteri* fermentation broth and *L. brevis* fermentation broth could effectively improve gut microbiota diversity and adjust the gut microbiota structure of HUA mice, and the effect was better than that of lactic acid bacteria fermentation supernatant. The studies have also found that *L. reuteri* L20M3 and *L. brevis* MJM60390 could improve gut microbiota disorders caused by hyperuricemia (20, 38). The intervention of LRS significantly reduced the diversity of gut microbiota in HUA mice, adversely affecting the recovery of the flora, which may be the reason why LR had a stronger effect on lowering uric acid than LRC. However, LBC not only contains *L. brevis* strains but also metabolites produced by the cells in the fermentation supernatant. *L. brevis* could increase the diversity of the gut microbiota of HUA mice, and its fermentation supernatant could improve intestinal metabolism. The combined effect of the two makes the uric acid lowering effect of LBC stronger than LBS. Studies have shown that the fermentation broth of LAB can regulate intestinal health, which may be caused by metabolites derived from fermentation such as acetic acid, butyric acid, lactic acid, acetaldehyde, and acetoin (39). The differences between LR, LRC, LBC, and LBS in regulating intestinal metabolism and gut microbiota may be caused by the effects of metabolites produced by the fermentation of lactic acid bacteria.

The relative abundance of *Firmicutes* was decreased in the HUA group, which was consistent with the results of Cao et al. (40). *Firmicutes* can participate in energy absorption by decomposing saturated fatty acids in the intestine. They are the main butyrate-producing bacteria, and these substances play an important role in intestinal health, such as providing nutrition to the intestinal mucosa (41). Therefore, the reduction of *Firmicutes* is not conducive to intestinal health. *Verrucomicrobia* was positively correlated with the occurrence of hyperuricemia, and the proportion of *Verrucomicrobia* was significantly increased in the HUA group. Ring and other researchers have found that the relative abundance of *Akkermansia* is positively correlated with the histopathology and the degree of colitis in mice with colitis and the abundance of *Akkermansia* in fecal samples of colorectal cancer patients significantly increased (42, 43). Hyperuricemia can induce intestinal inflammation, and the relative abundance of *Akkermansia* in the HUA group significantly increased, which was similar to the above research results. *Lactobacillus* and *Pseudomonas* can synthesize uric acid metabolic enzymes such as uric acid oxidase, allantoinase and allanicidase, which can sequentially degrade uric acid into 5-hydroxyisourate, allantoin, allantoic acid and urea (44). *Lachnospiraceae_FCS020_group* can produce beneficial metabolites for the host, such as butyrate. The relative abundance of these two bacteria in HUA group was significantly reduced. Supplementing *L. reuteri*, *L. reuteri* fermentation broth, *L. brevis* fermentation broth and *L. brevis* fermentation supernatant could increase the relative abundance of *Firmicutes*, *Lactobacillus*, and *Lachnospiraceae_FCS020_group* in HUA mice to varying degrees, and reduce the relative abundance of *Verrucomicrobia* and *Akkermansia*. It can improve the composition and structure of intestinal microorganisms, increase the abundance of beneficial bacteria and reduce the abundance of harmful bacteria. Ni et al. (38) have also found that after *L. reuteri* L20M3 intervention, the abundance of *Firmicutes* and *Lactobacillus* in the gut microbiota of HUA mice increased. Gut microbiota can affect changes in intestinal metabolites and thus affect body health. We used PICRUSt2 software to predict the metabolic function of gut microbiota in mice. The intervention of LR, LRC, LBC, and LBS changed the metabolic function of the gut microbiota in HUA mic and reversed the changes in metabolic pathways such as amino acid metabolism, terpene and polyketide metabolism, and energy metabolism. This may play a certain role in promoting the recovery of metabolic pathways such as amino acid metabolism and energy metabolism in intestinal metabolism.

## 5 Conclusion

This study found that the potential mechanism of *L. reuteri*, *L. reuteri* fermentation broth, *L. brevis* fermentation broth and *L. brevis* fermentation supernatant in relieving hyperuricemia may be related to repair intestinal metabolic disorders and improve the structure of gut microbiota. This study provides new inspiration for exploring their mechanism of action. However, the mechanism of alleviating hyperuricemia by lactic acid bacteria and their fermentation broth involves multiple targets and multiple channels, which has not been clearly clarified in this study. In the future, multi-omics, fecal microbiota transplantation and other techniques can be used to further clarify their specific components and targets for lowering uric acid.

## Data Availability

The datasets presented in this study can be found in online repositories. The names of the repository/repositories and accession number(s) can be found: https://www.ncbi.nlm.nih.gov/, PRJNA1159981.
