# Supplementary material for: Study on the mechanism of lactic acid bacteria and their fermentation broth in alleviating hyperuricemia based on metabolomics and gut microbiota
Source: Front Nutr. 2024 Dec 4;11:1495346. doi: 10.3389/fnut.2024.1495346 (PMC11652139; doi:10.3389/fnut.2024.1495346)
Supplement: Supplementary file 1 [file Data_Sheet_1.docx]

**
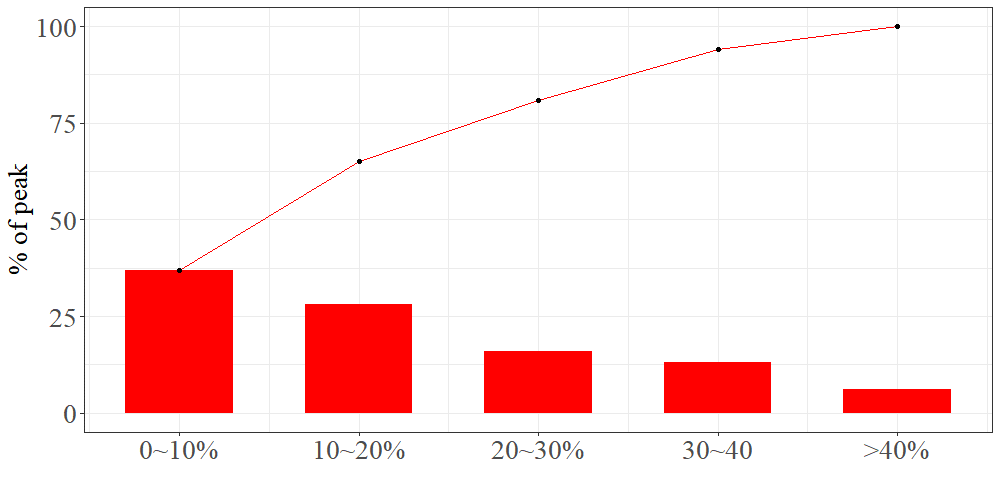
**

**Supplementary Figure 1.** The RSD distribution of feature peaks.

**Supplementary table 1.** The body weights of mice

| Group | 1 day (g) | 5 day (g) | 9 day (g) | 13 day (g) | The change rate of body weight （%） |
| --- | --- | --- | --- | --- | --- |
| CON | 34.38±0.74^abc^ | 38.63±1.24^ab^ | 41.00±0.96 ^ab^ | 42.51±1.60^ab^ | ﹢23.64 |
| HUA | 34.86±2.42^a^ | 38.75±2.57^a^ | 41.38±1.28^a^ | 43.38±2.13^a^ | ﹢24.37 |
| MRS | 34.75±1.04^ab^ | 37.38±0.91^abcd^ | 39.25±2.31^bcd^ | 39.38±3.20^d^ | ﹢13.31 |
| ALP | 34.51±1.93^abc^ | 34.38±1.28^e^ | 29.17±2.14^e^ | 32.43±5.29^f^ | -6.00 |
| LRC | 33.88±1.12^abc^ | 36.63±1.69^bc^ | 38.00±1.69^cd^ | 39.63±2.00^d^ | ﹢16.97 |
| LR | 33.51±1.60^abc^ | 35.88±0.99^cde^ | 37.50±1.58^cd^ | 39.33±2.94^d^ | ﹢17.41 |
| LRS | 33.01±1.07^abc^ | 36.00±1.54^cde^ | 38.00±1.20^cd^ | 37.43±3.51^e^ | ﹢13.41 |
| LBC | 33.63±2.20^abc^ | 37.13±3.64^abcd^ | 39.43±0.86^bcd^ | 40.71±2.63^bcd^ | ﹢21.08 |
| LB | 34.50±0.93^abc^ | 37.63±2.54^abc^ | 39.43±1.31^bcd^ | 42.13±2.30^abc^ | ﹢22.10 |
| LBS | 34.25±2.38^abc^ | 35.63±0.77^de^ | 39.00±1.54^cd^ | 41.01±3.11^bcd^ | ﹢19.71 |
| PLC | 32.88±1.36^bc^ | 36.29±1.23^bc^ | 39.14±2.81^bcd^ | 40.57±3.15^cd^ | ﹢23.41 |
| PL | 34.38±1.77^abc^ | 37.63±1.47^abc^ | 39.63±2.6^abc^ | 42.00±3.66^abc^ | ﹢22.18 |
| PLS | 32.75±1.49^bc^ | 36.75±2.1^bcd^ | 38.43±1.47^cd^ | 39.57±2.44^d^ | ﹢20.83 |

Note: Different letters indicate significant differences between groups (P<0.05).

**Supplementary table 2.** Biomarkers related to hyperuricemia

| Metabolites | HUA vs CON | ALP vs HUA | LB vs HUA | LBC vs HUA | LBS vs HUA | LR vs HUA | LRC vs HUA | LRS vs HUA |
| --- | --- | --- | --- | --- | --- | --- | --- | --- |
| Succinic acid | ↓ | —— | ↓ | —— | —— | ↑ | —— | —— |
| 2-Hydroxyskatole | ↓ | —— | —— | —— | —— | —— | —— | —— |
| L-Phenylalanine | ↓ | —— | —— | —— | —— | ↑ | —— | —— |
| Acetic acid | ↓ | —— | —— | —— | —— | ↑ | —— | —— |
| Penicillamine | —— | —— | ↑ | —— | —— | —— | —— | —— |
| Stearic acid | ↓ | —— | —— | —— | —— | —— | —— | —— |
| Uracil | ↑ | —— | —— | —— | —— | —— | —— | —— |
| Urea | —— | —— | ↑ | ↑ | ↑ | —— | —— | —— |
| Dopamine | ↓ | —— | —— | —— | —— | —— | —— | —— |
| L-Arginine | ↓ | —— | —— | —— | —— | —— | —— | —— |
| L-Leucine | —— | —— | ↓ | —— | —— | —— | —— | ↓ |
| Creatinine | ↓ | —— | —— | —— | —— | —— | ↓ | ↓ |
| Stearic acid | ↓ | —— | —— | —— | ↑ | ↑ | —— | ↑ |
| Uridine | ↓ | ↓ | —— | —— | ↓ | ↓ | —— | —— |
| L-Iditol | ↓ | —— | ↑ | —— | —— | ↓ | ↑ | —— |
| Dihydrouracil | ↑ |  | —— | —— | ↑ | —— | ↓ | —— |
| Purin | ↑ | ↓ | —— | —— | —— | —— | —— | ↓ |
| L-Glycine | ↓ | —— | —— | ↑ | —— | ↓ | ↑ | ↑ |
| Xanthine | ↑ | —— | —— | —— | ↓ | —— | —— | —— |
| alpha-Hydroxyisobutyric acid | ↑ | —— | —— | —— | —— | —— | —— | —— |
| Inosine | ↑ | —— | ↓ | —— | —— | —— | —— | —— |
| 24-Epicampesterol | ↓ | —— | ↓ | —— | —— | ↑ | —— | —— |
| Deoxycholic Acid | ↓ | —— | —— | ↓ | —— | —— | —— | —— |
| Oxalic acid | —— | ↑ | ↑ | —— | —— | —— | —— | —— |
| Hydroxypropanedioic acid | —— | ↑ | ↑ | —— | —— | —— | —— | —— |
| Diethanolamine | —— | ↑ | ↑ | ↑ | —— | —— | ↑ | —— |
| Glycerol | —— | ↑ | —— | —— | —— | —— | —— | —— |
| L-Methionine | —— | ↑ | —— | —— | —— | —— | —— | ↑ |
| Hypoxanthine | —— | ↑ | —— | ↑ | —— | —— | —— | —— |
| 4-Ethylbenzaldehyde | —— | ↑ | —— | —— | —— | —— | —— | ↑ |
| beta-Sitosterol | —— | ↑ | —— | —— | —— | —— | —— | —— |
| Oleic acid | —— | ↓ | ↑ | ↑ | —— | ↑ | ↓ | ↓ |
| Linoleic acid | —— | ↑ | —— | ↑ | —— | —— | —— | —— |
| 4-Isothiocyanato-1-butene | —— | —— | ↑ | —— | —— | —— | —— | —— |
| 3-Methyladenine | —— | —— | ↓ | —— | —— | —— | —— | —— |
| Dopamine | —— | —— | ↑ | —— | —— | —— | —— | —— |
| Succinic acid, 3-chlorophenyl 2-fluoroethyl ester | —— | —— | ↑ | —— | —— | —— | —— | —— |
| L-Tyrosine | —— | —— | ↑ | ↑ | —— | —— | —— | —— |
| 1-O-Tetradecylglycerol | —— | —— | ↑ | —— | —— | —— | ↑ | —— |
| Menthol | —— | —— | ↑ | —— | —— | —— | —— | —— |
| beta-Sitosterol | —— | —— | ↓ | —— | —— | —— | ↑ | —— |
| Stigmasterol | —— | —— | ↑ | ↑ | —— | —— | —— | —— |
| 5-Aminohexanoic acid | —— | —— | ↑ | —— | —— | —— | —— | —— |
| L-Threonine | —— | —— | —— | ↑ | —— | ↑ | —— | —— |
| d-Mannose | —— | —— | —— | ↓ | —— | —— | —— | —— |
| L-Tryptophan, 1-(trimethylsilyl)-, trimethylsilyl ester | —— | —— | —— | ↑ | —— | —— | —— | ↑ |
| L-Alanine | —— | —— | —— | —— | ↓ | ↑ | —— | —— |
| D-Malic acid | —— | —— | —— | —— | ↓ | —— | ↓ | —— |
| L-Proline | —— | —— | —— | —— | ↓ | —— | —— |  |
| L-Cysteine | —— | —— | —— | —— | ↑ | —— | —— |  |
| Methyl α-D-ribofuranoside | —— | —— | —— | —— | ↓ | —— | —— | ↓ |
| α-Tocopherol | —— | —— | —— | —— | ↓ | —— | —— | ↓ |
| Cholesterol | —— | —— | —— | —— | ↑ | —— | —— | —— |
| Tryptamine | —— | —— | —— | —— | —— | ↑ |  | —— |
| Glycolic acid | —— | —— | —— | —— | —— | ↑ | ↑ | —— |
| Methylmalonic acid | —— | —— | —— | —— | —— | ↑ | —— | —— |
| L-Valine | —— | —— | —— | —— | —— | ↓ | —— | ↓ |
| Sarcosine | —— | —— | —— | —— | —— | ↑ | —— | ↓ |
| Phenylpyruvic acid | —— | —— | —— | —— | —— | ↑ | ↑ | —— |
| Putrescine | —— | —— | —— | —— | —— | ↑ | —— | —— |
| 2-Hydroxyskatole | —— | —— | —— | —— | —— | —— | ↓ | —— |
| 2-Methylcyclohexanone | —— | —— | —— | —— | —— | —— | ↓ | —— |
| L-Serine | —— | —— | —— | —— | —— | —— | ↑ | ↓ |
| Succinic acid | —— | —— | —— | —— | —— | —— | —— | ↑ |
| L-Glutamic acid | —— | —— | —— | —— | —— | —— | —— | ↑ |
| Trehalose | —— | —— | —— | —— | —— | —— | —— | ↓ |
| L-Lysine | —— | —— | —— | —— | —— | —— | —— | ↑ |

↑: indicates up-regulation of metabolites; ↓: indicates down-regulation of metabolites; ——: indicates no significant difference in the metabolites
